# Supplementary material for: Four decades of genomic stability and adaptive divergence in Xanthomonas phages: defining Duraznoxanthovirus arenicola and its evolutionary framework
Source: Front Microbiol. 2026 Apr 29;17:1779411. doi: 10.3389/fmicb.2026.1779411 (PMC13168109; doi:10.3389/fmicb.2026.1779411)
Supplement: Supplementary file 1 [file Table_1.docx]

**STable 1.** Summary of the 30 publicly available *Phytobacteriaviridae* phage genomes as of October 27^th^, 2025.

| **Phage** | **Bacterial Host** | **Phage Genus** | **Length (bp)** | **NCBI GenBank Accession Number** |
| --- | --- | --- | --- | --- |
| Bcep1 | *Burkholderia cepacia* | *Naesvirus* | 48,177 | NC_005263.2 |
| Bcep43 | *Burkholderia cepacia* | *Naesvirus* | 48,024 | NC_005342.2 |
| Bcep781 | *Burkholderia cepacia* | *Naesvirus* | 48,247 | NC_004333.2 |
| BcepNY3 | *Burkholderia cenocepacia* | *Naesvirus* | 47,382 | NC_009604.1 |
| Adzire | *Ralstonia pseudosolanacearum* | *Bakolyvirus* | 44,732 | NC_054462.1 |
| Bakoly | *Ralstonia pseudosolanacearum* | *Bakolyvirus* | 44,222 | NC_054463.1 |
| Elie | *Ralstonia pseudosolanacearum* | *Bakolyvirus* | 44,966 | MT740735.1 |
| Jenny | *Ralstonia pseudosolanacearum* | *Bakolyvirus* | 43,921 | MT740744.1 |
| Sarlave | *Ralstonia pseudosolanacearum* | *Bakolyvirus* | 44,858 | MT740746.1 |
| Simangalove | *Ralstonia pseudosolanacearum* | *Bakolyvirus* | 44,834 | NC_054946.1 |
| RS_CA1 | *Ralstonia solanacearum* & *Ralstonia pseudosolanacearum* | Unclassified | 46,254 | PP316169.1 |
| PQ43W | *Ralstonia pseudosolanacearum* | Unclassified | 47,156 | PP405626.1 |
| BsXeu269p3 | *Xanthomonas euvesicatoria* | *Beograduvirus* | 46,280 | ON996340.1 |
| KPhi1 | *Xanthomonas euvesicatoria* | *Beograduvirus* | 46,077 | NC_054460.1 |
| MYK3 | *Xanthomonas* spp. | *Beograduvirus* | 47,500 | OK275494.1 |
| phiXaf18 | *Xanthomonas vesicatoria* | *Beograduvirus* | 47,407 | NC_054461.1 |
| pXoo2107 | *Xanthomonas oryzae* pv. *oryzae* | *Tsukubavirus* | 46,052 | OP067662.1 |
| X2 | *Xanthomonas oryzae* pv. *oryzae* | *Tsukubavirus* | 45,966 | MW435566.1 |
| OP2 | *Xanthomonas oryzae* pv. *oryzae* | *Tsukubavirus* | 46,643 | NC_007710.1 |
| XPV1 | *Xanthomonas oryzae* pv. *oryzae* | *Tsukubavirus* | 46,503 | NC_054459.1 |
| XPV2 | *Xanthomonas oryzae* pv. *oryzae* | *Tsukubavirus* | 45,969 | MG944235.1 |
| XPV3 | *Xanthomonas oryzae* pv. *oryzae* | *Tsukubavirus* | 47,046 | MG944236.1 |
| XPP1 | *Xanthomonas oryzae* pv. *oryzae* | *Tsukubavirus* | 46,195 | NC_054458.1 |
| XPP2 | *Xanthomonas oryzae* pv. *oryzae* | *Tsukubavirus* | 46,480 | MG944228.1 |
| XPP3 | *Xanthomonas oryzae* pv. *oryzae* | *Tsukubavirus* | 49,612 | MG944229.1 |
| XPP4 | *Xanthomonas oryzae* pv. *oryzae* | *Tsukubavirus* | 47,397 | MG944230.1 |
| XPP6 | *Xanthomonas oryzae* pv. *oryzae* | *Tsukubavirus* | 46,281 | MG944231.1 |
| XPP8 | *Xanthomonas oryzae* pv. *oryzae* | *Tsukubavirus* | 46,278 | MG944232.1 |
| XPP9 | *Xanthomonas oryzae* pv. *oryzae* | *Tsukubavirus* | 48,669 | MG944233.1 |
| NEB7 | *Xanthomonas* spp. | Unclassified | 45,241 | OQ676962.1 |
