# Supplementary material for: Four decades of genomic stability and adaptive divergence in Xanthomonas phages: defining Duraznoxanthovirus arenicola and its evolutionary framework
Source: Front Microbiol. 2026 Apr 29;17:1779411. doi: 10.3389/fmicb.2026.1779411 (PMC13168109; doi:10.3389/fmicb.2026.1779411)
Supplement: Supplementary file 2 [file Table_2.docx]

**
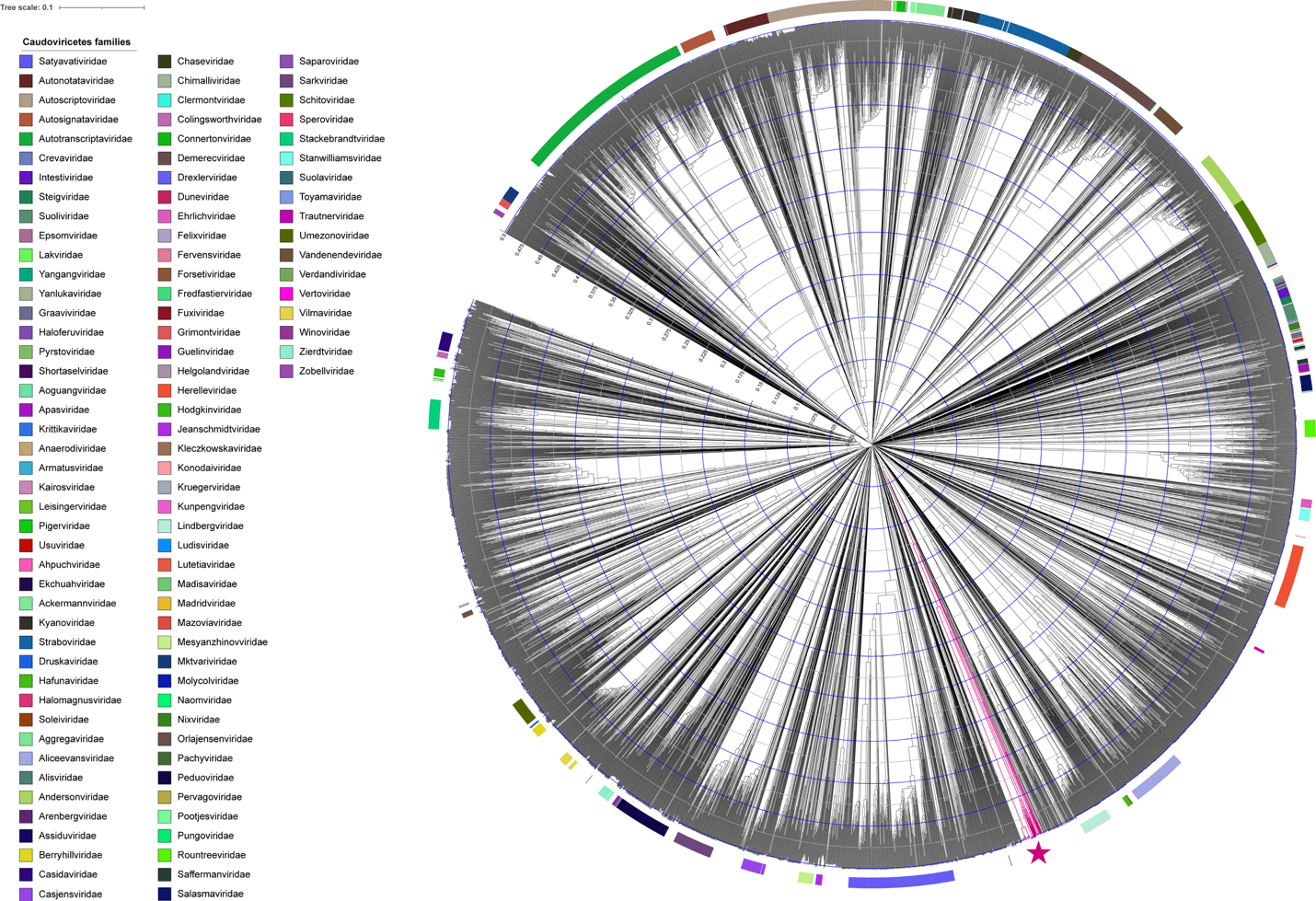
**

**Figure S1.** Viral proteomic tree (ViPTree) constructed from 5,822 reference genomes, with the 15 Xapϕ phages infecting *Xanthomonas arboricola* pv. *pruni* (Xap) highlighted in magenta and marked with a star. Taxonomic family assignments for reference genomes are indicated by the color of the outer bar.


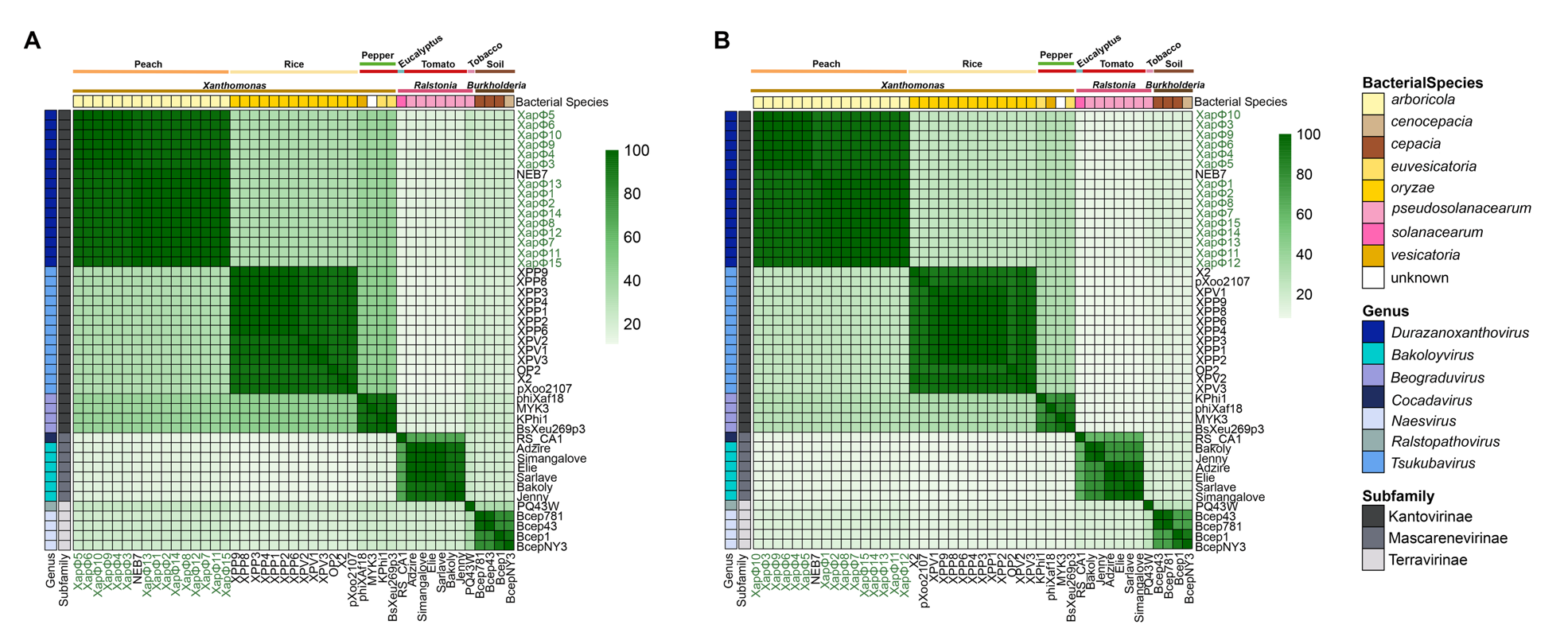


**Figure S2.** Genomic and proteomic similarity analyses reveal a novel phage clade: *Duraznoxanthovirus arenicola*. (A) Heatmap of VIRIDIC-calculated intergenomic distances among Xapϕ1–Xapϕ15 and 30 closely related *Phytobacteriaviridae* phages. The Xapϕ1–Xapϕ15 cluster, in conjunction with phage NEB7, forms a distinct, *Duraznoxanthovirus arenicola*. (B) Heatmap of Jaccard similarity index values based on protein identity shared among Xapϕ1–Xapϕ15 and 30 closely related *Phytobacteriaviridae* phages. Phage genus and subfamily are color-coded as indicated by the bar on the left, with the key on the right. The bacterial host genus, species, and isolation source are listed at the top of each heatmap.


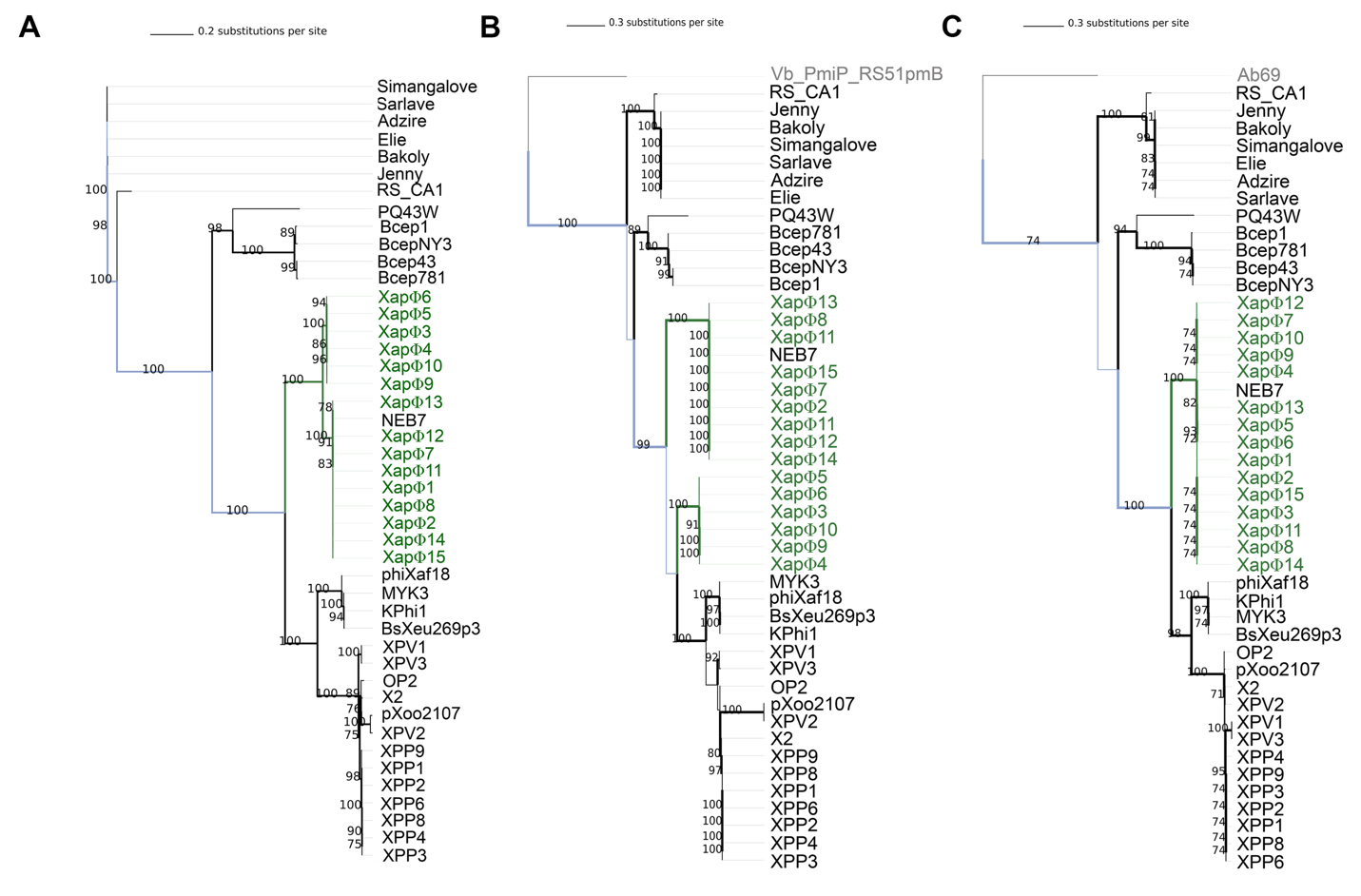


**Figure S3.** Phylogenetic analyses of *Duraznoxanthovirus arenicola* phages reveal evolutionary relationships within Phytobacteriaviridae. (A) Midpoint-rooted maximum likelihood phylogenetic tree of Xapϕ1–Xapϕ15 and 30 closely related Phytobacteriaviridae phages, inferred from a core genome alignment of ten conserved genes using IQ-TREE. Maximum likelihood phylogenetic tree of Xapϕ1–Xapϕ15 and 30 closely related Phytobacteriaviridae phages, inferred from an amino acid alignment of the (B) TerL sequence and (C) the portal protein sequence using IQ-TREE. The TerL sequence from Proteus phage vB_PmiP_RS51pmB (QDH85558.1) and the portal protein sequence from Acinetobacter phage Ab69 (WMC00310.1) were used as outgroups, respectively.
